# Influence of extracellular oscillations on neural communication: a computational perspective

**DOI:** 10.3389/fncom.2014.00009

**Published:** 2014-02-07

**Authors:** Zoran Tiganj, Sylvain Chevallier, Eric Monacelli

**Affiliations:** LISV, Université de Versailles Saint-Quentin-en-YvelinesVélizy-Villacoublay, France

**Keywords:** extracellular oscillations, local field potentials, ephaptic coupling, nonsynaptic communication, multi-compartment model, NEURON simulation environment, pyramidal neurons

## Abstract

Neural communication generates oscillations of electric potential in the extracellular medium. In feedback, these oscillations affect the electrochemical processes within the neurons, influencing the timing and the number of action potentials. It is unclear whether this influence should be considered only as noise or it has some functional role in neural communication. Through computer simulations we investigated the effect of various sinusoidal extracellular oscillations on the timing and number of action potentials. Each simulation is based on a multicompartment model of a single neuron, which is stimulated through spatially distributed synaptic activations. A thorough analysis is conducted on a large number of simulations with different models of CA3 and CA1 pyramidal neurons which are modeled using realistic morphologies and active ion conductances. We demonstrated that the influence of the weak extracellular oscillations, which are commonly present in the brain, is rather stochastic and modest. We found that the stronger fields, which are spontaneously present in the brain only in some particular cases (e.g., during seizures) or that can be induced externally, could significantly modulate spike timings.

## 1. Introduction

Ion exchange between neurons and the surrounding extracellular medium is essential for neural communication. In the extracellular medium, this exchange causes oscillations of electric potential, commonly referred to as Local Field Potentials (LFPs) (Ebersole and Milton, 2003; Buzsáki et al., 2012). The LFPs often exhibit significant levels of temporal and spatial coherence and are linked with a number of cognitive phenomena (see Ward 2003; Buzsáki and Draguhn 2004; Uhlhaas et al. 2010; Wang 2010 for reviews). For instance, theta and gamma oscillations, particularly in the hippocampus, are often linked with memory formation and retrieval (Schack et al., 2002; Sederberg et al., 2003; Osipova et al., 2006; Nyhus and Curran, 2010), while attention is associated with a reduced alpha rhythm and increased gamma rhythm (Thut et al., 2006; Lisman and Buzsáki, 2008; Capotosto et al., 2009; Deco and Thiele, 2009; Schroeder and Lakatos, 2009). Different rhythms are related with processing in early sensory systems (Koepsell et al., 2010) and in speech generation and processing (Peelle and Davis, 2012). Furthermore, it has recently been proposed that some neurons code information by firing with respect to the phase of the ongoing oscillations (Fries et al., 2007; Montemurro et al., 2008; Kayser et al., 2009).

The extracellular oscillations are commonly considered to be an epiphenomena, a sporadic consequence of neural dynamics. Contrary to this hypothesis, it has been speculated that the periodic oscillations in the extracellular medium in some cases have a functional role, that they support the neural communication. This alternative hypothesis, based on *ephaptic transmission*, assumes that the neurons might not communicate only by exchanging information through their synaptic connections, but additionally through the extracellular medium (Fröhlich and McCormick, 2010; Weiss and Faber, 2010; Anastassiou et al., 2011). Even though the LFPs are generally too weak to trigger an action potential, they can still affect the spike number and timing (Parra and Bikson, 2004; Radman et al., 2007; Anastassiou et al., 2010).

If the hypotheses about the functional role of the extracellular oscillations are proven right, it will provide new insights for understanding the neural code, indicating mechanisms that can contribute to the widely observed synchrony of the neural activity. This finding could have impact on theoretical and clinical research, including neuropharmacology and neuroprostetics. Additionally, it would be beneficial for developing new approaches in computational models of the brain, and more generally, models of artificial neural networks, since the existing models (e.g., Izhikevich, 2006; Markram, 2006) do not address the ephaptic transmission.

The main motivations for the proposed study are to deepen the understanding of this prospective mode of neural communication and to investigate whether the externally induced electrical fields (e.g., through Transcranial Magnetic Stimulation (TMS) (Barker et al., 1985; Hallett, 2000; Walsh and Cowey, 2000; Allen et al., 2007), Transcranial Electrical Stimulation (TEC) (Deans et al., 2007; Kirov et al., 2009; Ozen et al., 2010; Reato et al., 2010) and Deep Brain Stimulation (DBS) (Benabid et al., 2009; Joucla and Yvert, 2012) might promote the ephaptic coupling. Modifying the ephaptic coupling could alter synchrony of the neural assemblies and potentially help to correct pathological network dynamics.

Through a computational study, we investigated what changes in the timing and number of action potentials can be induced by the temporal and spatial oscillations of the extracellular potential. Our study is conducted at a single neuron level and is done through several simulation setups, hard to implement experimentally. Contrary to the common experimental protocols, where the stimulation is done by current injection into the soma (see for example Anastassiou et al. 2011), we used PostSynaptic Potentials (PSPs) which are applied at different spatial locations of the dendrites. We generated a sufficient number of PSPs for the neuron to exhibit different firing patterns. Moreover, we analyzed the influence of extracellular oscillations with respect to the spatial part of the dendritic tree receiving most of the inputs. We modeled the dendrites with active ion conductances and considered different dendritic morphologies. Since the considered neuronal model is multi-compartmental, we computed the strength of the extracellular oscillations for each compartment separately, so that it depends on the spatial location of the compartment.

Focusing on models of the CA1 and the CA3 neurons, we investigated the influence of the extracellular oscillations at a single neuron level depending on:

1. Properties of the active ion conductances, i.e., we used different sets of active ion channels.
2. Spatial distribution of the dendritic inputs, i.e., we altered the locations of the synaptic inputs between the basal and apical branches.
3. Morphological properties, i.e., we used different shapes of the dendritic tree.

**Figure 1:**
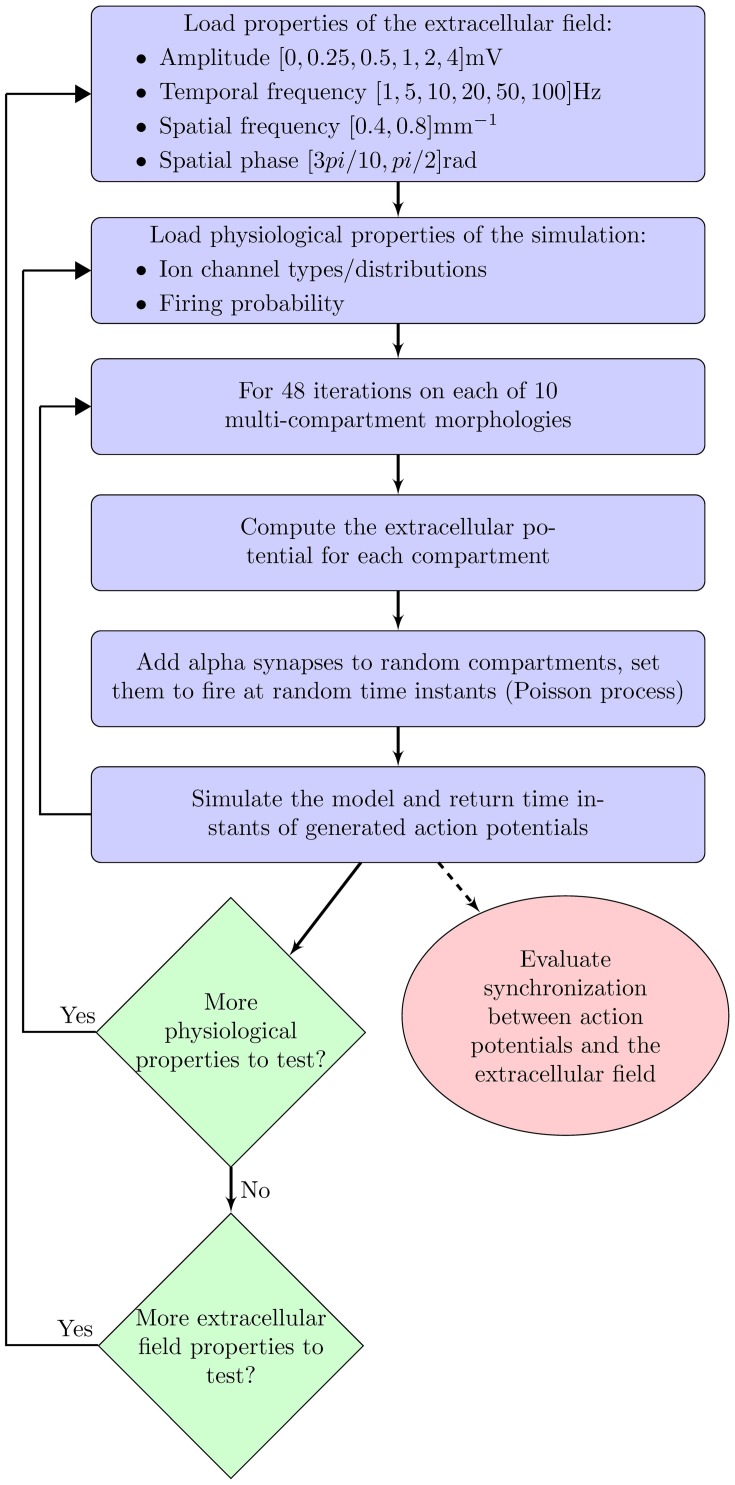
**Flowchart of the computational model**.

For each of these three cases, we examined the following properties of the extracellular oscillations (as shown in Figure 1): the temporal and spatial frequencies, the amplitude, and the phase. In this study, we always addressed the influence of the oscillations on an individual neuron, not on neural assemblies. Through randomly delivered PSPs the membrane potential was set to fluctuate around the threshold level, with a certain probability of crossing it. We studied how the above properties of the extracellular oscillations influence the number and the timing of generated spikes.

We did not investigate directly the influence of the spontaneous LFPs or the artificially induced oscillations, but the influence of the extracellular oscillations on a more general level, regardless of their origin. The link between this general level and the spontaneous LFPs of a particular temporal and spatial properties can still be made and will be discussed here.

It is important to mention that the proposed study is difficult to perform experimentally, either *in vivo* or *in vitro*. It is particularly difficult to produce repetitive stimulation with the PSPs of controlled amplitude, timing and spatial distribution. Since such repetitive stimulation is essential for our investigation, we find it suitable to approach the problem through a computational study. Complex interactions of nonlinear ionic currents within branched dendritic trees can be described through a set of equations, based on the Hodgkin–Huxley model, which cannot be solved analytically. We used the simulator NEURON (Hines and Carnevale, 1997) to numerically solve the equations (Mainen and Sejnowski, 1998). This study is not a replacement for *in vivo* or *in vitro* experiments, but rather a complementary approach that can help in focusing future research.

Common use of the Hodgkin–Huxley model assumes that the extracellular medium is equipotential and it is typically considered as an electrical ground. Here we assume that the electric potential in the extracellular space varies through time and space. If we consider the neural membrane to consist of the ohmic conductance (active and passive) and the capacitance, the above assumption implies that the voltage on the conductance and capacitance is not simply equal to the intracellular membrane potential, which is the case in the Hodgkin–Huxley model. Instead, it is set to be equal to the difference between the intracellular membrane potential and the instantaneous amplitude of the extracellular oscillations.

The paper is organized as follows. Parameters of the model are closely analyzed in the following section. In particular, we describe the neural morphologies and give details on the properties of the ion channels in the model. We also describe the model of PSPs and the extracellular oscillations. In section 3 we present the results of the simulations: first, when the stimulation is done with the current injection, and then when it is done with the PSPs. The results illustrate the importance of the amplitude, the temporal and spatial frequency and the spatial phase of the extracellular oscillations. Finally, the results are discussed in section 4.

## 2. Materials and methods

This section provides details on the computational models that are used to obtain the results presented in the paper. The models are implemented in neural simulator NEURON (Hines and Carnevale, 1997) that we run on a Linux-based cluster, consisting of 75 dual-core computers. We used multi-compartment models for two different neuron types: the CA1 and the CA3 rat hippocampal pyramidal neurons. The time step of the simulation was 25μs. The stability of the models with respect to the time step was tested by verifying that for smaller times steps the influence of the extracellular oscillations does not change significantly. To account for the artifacts caused by the initial conditions we added an extra period of 100 ms at the beginning of each simulation (not displayed in the results) that allowed the membrane potential to grow from the initial −65 mV to the value controlled only by the PSPs.

### 2.1. Morphology

Each neuron in our model consists of the dendritic tree, the soma and the short axon. The length of the axon was determined experimentally (100μm), in a such way that simulating longer axon did not quantitatively change the results. For each of the two neuron types, we investigated five different morphologies of the dendritic tree. The morphologies of the CA3^1^ are taken from Samsonovich and Ascoli (2003) and those of the CA1 cells^2^ are taken from Pyapali et al. (1998).

All the morphology files are given in SWC format. In this format, each segment is described as a cylinder and is associated with a type (soma, axon, basal, or apical dendrite), a spatial position of the end point, a diameter, and the information about which segment is connected to the given one on the path to the soma. Thus, from such files we were able to precisely reconstruct 3D shapes of the given morphologies. Each morphology file describes a branched dendritic tree, with the number of compartments spanning from 151 to 503. The size of the compartments was computed using the d_lambda rule proposed by Carnevale and Hines (2006).

### 2.2. Properties of the active ion conductances

To study how the influence of the extracellular oscillations on the spike number and timing depends on the active ion conductances in the soma and the dendrites, two different sets of active ion conductances were used, one corresponding to the CA1 and the other to the CA3 pyramidal neurons. Notice that adding active conductances in the dendrites was necessary for the PSPs to propagate in a significant amount from the apical dendrites to the soma. In such way the PSPs could contribute to the generation of the dendritic as well as the somatic spikes (Kim et al., 2012). Properties of the ion channels used in the simulations have been described in Migliore et al. (2005) for the CA1 neurons and in Hemond et al. (2008) for the CA3 neurons^3^:

- CA1: Uniform passive properties were τ_*m*_ = 28 ms (membrane time constant), *R*_*m*_ = 28 kΩcm^2^ (radial resistance of the membrane), *R*_*a*_ = 150 Ωcm (axial resistance of the membrane), while the active properties included sodium, DR-, and A-type potassium conductances and a hyperpolarization-activated *I*_*h*_ current. The kinetics for all the channels and the spatial distribution of the channels were identical to those in Migliore et al. (2005).
- CA3: Passive: τ_*m*_ = 35 ms, *R*_*m*_ = 25 Ωcm^2^, *R*_*a*_ = 150 Ωcm. The set of active channel properties included sodium conductances, a repertoire of potassium conductances: delayed rectifier, M-current, fast-inactivating A-type, and slowly-inactivating D-type current, three voltage-gated Ca^2+^ conductances (Ca_*V*_ N-, L-, and T-type), two calcium-dependent potassium conductances (KC and KAHP), and *I*_*h*_ current.

These two sets give rather different profiles of ion conductances. While for the CA1 neurons only the main conductances involved in the action potential generation and regulation were included, for the CA3 neurons we included a much broader set of ion conductances.

### 2.3. Model of postsynaptic potentials

The spatial location of each synapse and their corresponding PSPs times were determined randomly for each simulation. We repeated each simulation for several different properties of the extracellular field, e.g., we varied the amplitude of the extracellular field. To study the influence of the extracellular oscillations, we kept the spatial locations and the PSPs times fixed for each different property of the extracellular field.

**Figure 2:**
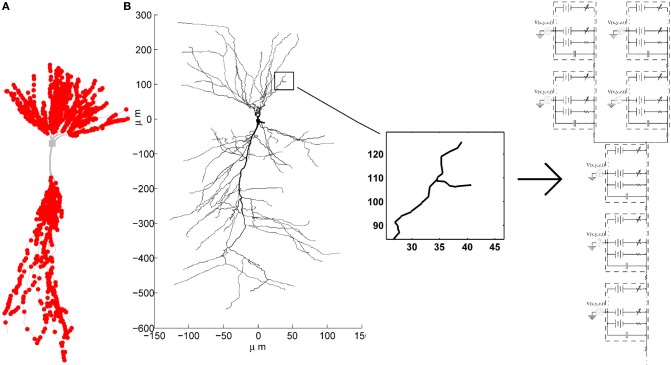
**(A)** Example of synaptic locations (red dots) on one of the CA3 neuron morphologies. **(B)** Multi-compartment model of a CA3 neuron with active conductances and the extracellular oscillations added to each compartment.

To emphasize spatiotemporal summation of the PSPs, we balanced the contributions from different synapses by pacing them only onto dendritic compartments thinner than 0.35μm. The probability of a synapse being placed onto any compartment thinner than 0.35μm was set to be equal, unless otherwise specified. The exact spatial location within the compartment was also determined randomly, with equal probability that the synapse will be placed onto any of the sections within the compartment. One example of synaptic locations generated in such way is given in Figure 2A.

Temporal profile of the PSPs was modeled as a Poisson process. The number of the PSPs per second was adjusted so that the neuron exhibits a firing rate *F* within a desired range. We define three ranges of the firing rate: low for *F* ⩽ 5 Hz, medium for 5 < *F* < 15 Hz and high for *F* ⩾ 15 Hz. To achieve these firing rates we altered the of the PSPs to be between 200 and 7000 Hz.

The synaptic conductance is modeled for *t* ⩾ *t*_onset_ as an alpha function:

(1) $$g=g_{\text{max}}\frac{t-t_{\text{onset}}}{\tau }e^{-\frac{1}{\tau }\left(t-t_{\text{onset}}-\tau \right)},$$

while for *t* < *t*_onset_, *g* = 0. The parameters within the alpha function were the same for all the synapses: τ = 0.1 ms, *g*_max_ = 1μS.

### 2.4. Model of the extracellular oscillations

**Figure 3:**
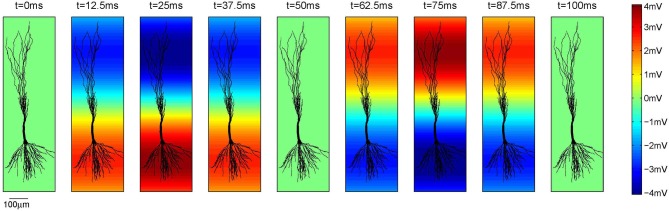
**The amplitude of the extracellular oscillations across one full temporal cycle of 100 ms, i.e., *f*_*t*_ = 10 Hz.** In this example *V*_*o*_ = 4 mV, *f*_*s*_ = 0.8 mm^−1^ and ϕ_*s*_ = $\frac{3\pi }{10}$ rad. The parameter ϕ_*s*_ is expressed at the bottom of the plots as the reference.

The voltage oscillations were simulated in the extracellular medium by adding a voltage source to each compartment^4^, as illustrated on Figure 2B. We modeled the oscillations as a temporal and spatial fluctuation. The change across space was only occurring along the somatodendritic axis, which we denote as the *x* axis. The other two axes of the Cartesian coordinate system are denoted as *y* and *z*. Consequently, at any time, the amplitude of the extracellular oscillations was different for different spatial points along the somatodendritic axis, but the same for any spatial point along the other two axis (*y* and *z*). This is depicted on a 2D plane in Figure 3. Shape of the oscillations is described by a simple sinusoidal function *V*_*e*_(*x*, *y*, *z*, *t*). Then, the extracellular potential at the spatial point (*x*, *y*, *z*) and at the time *t* writes as follows:

(2) $$V_{e}(x,y,z,t)=V_{o}\sin(2\pi f_{t}t)\sin(2\pi f_{s}x+\phi_{s})$$

where *V*_*o*_ is the amplitude of the oscillations, *f*_*t*_ is the temporal frequency, *f*_*s*_ is the spatial frequency and ϕ_*s*_ is the spatial phase.

In the context of the Hodgkin–Huxley model, the equation for the current *I*_*k*_ going through the *k*th ion channel is:

(3) $$I_{k}=g_{k}(V_{i}-V_{e}-V_{k})$$

where *V*_*i*_ is the intracellular potential, *V*_*k*_ is the reversal potential of the *k*th ion channel and *g*_*k*_ is the channel conductance.

To have better understanding of the influence of the extracellular oscillations on neural activity, the simulations were repeated for different values of *V*_*o*_, *f*_*t*_, *f*_*s*_ and ϕ_*s*_.

The amplitude of the extracellular oscillations depends on many factors, from the animal species, to the regions of the brain and the cognitive states (Buzsáki et al., 2012). In the band below 100 Hz, the amplitude rarely exceeds 1 mV (Anastassiou et al., 2010; Brette and Destexhe, 2011; Buzsáki et al., 2012). We performed the simulations for the following set of amplitudes *V*_*o*_: 0.25, 0.5, 1, 2 and 4 mV. This wide range of values allowed us to investigate the significance of the amplitude in a rather general context, including the spontaneous and the artificially induced oscillations.

The extracellular oscillations that exhibit a significant spatial extent commonly have temporal frequencies below 100 Hz, although there are notable exceptions, such as the hippocampal sharp waves (Buzsáki, 1986). Even though the high amplitude oscillations are known to occur in several particular bands, e.g., theta waves (Kahana et al., 2001), we performed the simulations for a relatively broad set of frequency values: 1, 5, 10, 20, 50, and 100 Hz. These values have been chosen to investigate whether the values observed in reality are in some sense optimal for high or low influence on neural activity.

**Figure 4:**
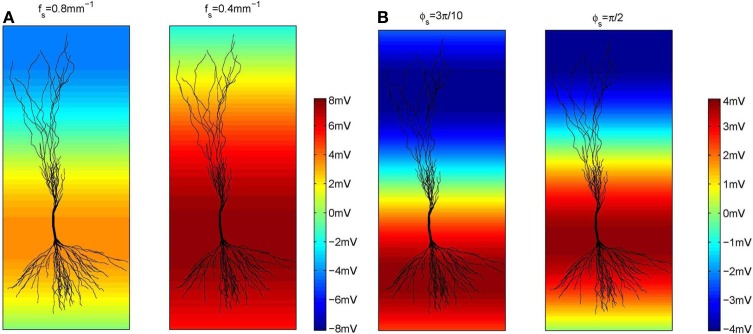
**(A)** The amplitude of the extracellular oscillations for two spatial frequencies, *f*_*s*_ = 0.8 mm (the left side plot) and 0.4 mm^−1^ (the right side plot). **(B)** The amplitude of the extracellular oscillations for two spatial phases, ϕ_*s*_ = $\frac{3\pi }{10}$ rad (left side plot) and $\frac{\pi }{2}$ rad (right side plot).

Regarding the spatial frequency, sources and sinks were located at the opposite sides of the dendritic tree, e.g., if the sources were located around the apical dendrites, then the sinks were located around the basal dendrites and vice versa. Therefore, we set *f*_*s*_ = 0.8 mm^−1^ as illustrated in Figure 3. The influence of the extracellular oscillations when sinks and sources were more distant from each other was also investigated by setting the spatial frequency *f*_*s*_ = 0.4 mm^−1^. In this case, to ensure the consistency of the results, the strength of the electrical field was kept constant: when the distance between the sink and the source was doubled, the amplitude of the extracellular oscillations was also doubled. The spatial distributions of the oscillations for both cases are shown in Figure 4A.

The spatial phase of the oscillations was set so that the peak amplitude was reached around the middle of the basal dendrites and around the upper end of the apical dendrites, as these areas often contain the largest number of branches. The exact value of the phase was ϕ_*s*_ = $\frac{3\pi }{10}$ rad, as shown on the left hand side plot in Figure 4B. Additionally, we investigated a case when the peak amplitude was reached around the soma. In that case ϕ_*s*_ = $\frac{\pi }{2}$ rad was used (see the right hand side plot in Figure 4B).

## 3. Results

In this section we first present the results of a simulation conducted with current injection as the stimulus input. We then present the results from a set of simulations in which the PSPs, instead of current injection, were used to stimulate the neuron.

### 3.1. Stimulation with current injection

**Figure 5:**
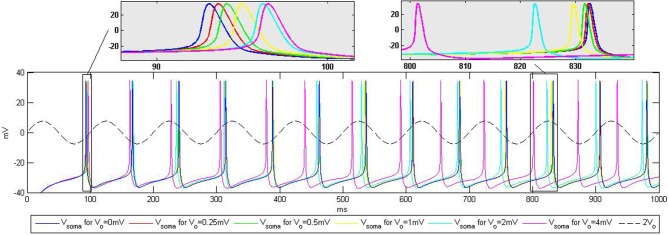
**Influence of the extracellular oscillations on the timing of the action potentials during current injection into the soma.** The top boxes highlight the influence of the extracellular oscillations on the spike timing. The baseline (no extracellular oscillations) is displayed in blue. The dashed line represents the extracellular oscillations. We display the maximum amplitude used in the simulations, *V*_*o*_ = 4 mV, scaled up by the factor of two for better visibility.

As an initial validation of the models used in this study we performed a simulation that is similar to the experimental protocol described in Anastassiou et al. (2011). The goal of the simulation was to analyze the influence of extracellular oscillations on spike timing during current injection into soma. The results are displayed in Figure 5.

For the CA3 neuron model, the injection of a 0.35 nA constant current into the soma led to a tonic spiking, such that each action potential was fired periodically, approximately every 75 ms. Adding the extracellular oscillations affected this periodicity, degrading the period stability. The time shift of the spikes increases with the amplitude of the oscillations. However, the increase is not linear, due to the complex nonlinear dynamics of the voltage-gated ion channels in the soma and in the dendritic tree.

The results displayed in Figure 5 are qualitatively similar to those reported in the experiments presented in Anastassiou et al. (2011). This type of simulation demonstrates that the extracellular oscillations can affect the timing of action potentials. Nevertheless, the influence of the low amplitude oscillations, which are observable in different cortical structures, seems to be rather modest. The temporal shift of the firing instants is very small, e.g., the influence of *V*_*o*_ = 0.25 mV oscillations results in 0.5 ms change in the timing of the action potential shown in the zoomed part on the top of Figure 5.

### 3.2. Stimulation with postsynaptic potentials

Current injection into the soma gives an illustration of the influence of extracellular oscillations on the timing of action potentials. However, this is not a very realistic setup and. Stimulation mediated by the PSPs is more biologically realistic. The PSPs properties were set as described in section 2.3 and the results of the simulation are presented for different properties of the extracellular oscillations according to the parameters presented in section 2.4.

#### 3.2.1. Influence of the amplitude of the extracellular oscillations

**Figure 6:**
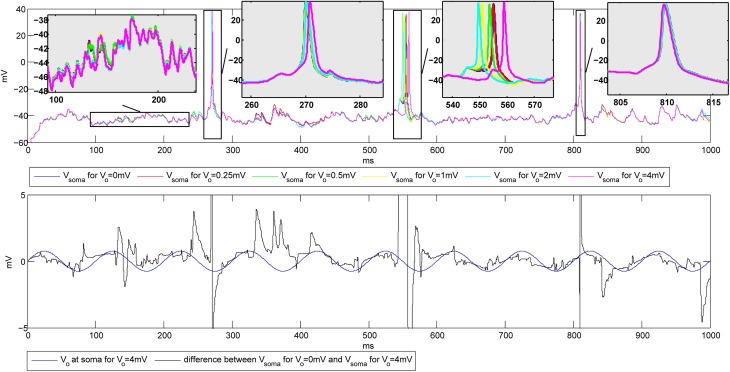
**Top: Influence of the extracellular oscillations on the timing of the action potentials during the stimulation with PSPs. Bottom:** The extracellular oscillations at the soma (*V*_*o*_ = 4 mV) and the difference between the membrane potential of the soma without and with the extracellular oscillations of 4 mV amplitude. The vertical lines on the **bottom** plot that exceed the plot boundaries results from action potentials visible on the **top** plot.

The simulations were first performed to explore the influence of the amplitude of the extracellular oscillations on the spike number and timing. The temporal frequency was set to 10 Hz, the spatial frequency to 0.8 mm^−1^ and the spatial phase to $\frac{-3\pi }{10}$ rad. The plots on the top of Figure 6 show the membrane potential at the soma of a CA3 neuron for different values of the amplitude of the oscillations. Hereafter we will refer to *baseline* as the evolution of the soma potential evoked by the PSPs in absence of the extracellular oscillations, i.e., it corresponds to the simulations with *V*_*o*_ = 0 mV. The baseline is shown in blue in Figure 6. In this example, the PSPs frequency was tuned so that the firing rate *F* stays below 5 Hz. The zooms of the four different parts of the plot illustrate the small changes of the membrane potential, as well as the small time shifts in the spike timing, both due to the influence of the extracellular oscillations.

The bottom plot in Figure 6 further illustrates how the membrane potential depends on the amplitude of the extracellular oscillations. The plot shows the membrane potential at the soma in the presence of 4 mV extracellular oscillations subtracted from the membrane potential at the soma with no extracellular oscillations (black line). To illustrate the phase relationship, on the same plot we also displayed the extracellular oscillations (blue line). The phase and amplitude of the difference of the two membrane potentials follow those of the extracellular oscillations, but due to a large nonlinearity of the voltage-gated channels, large fluctuations of the amplitude occur frequently.

**Figure 7:**
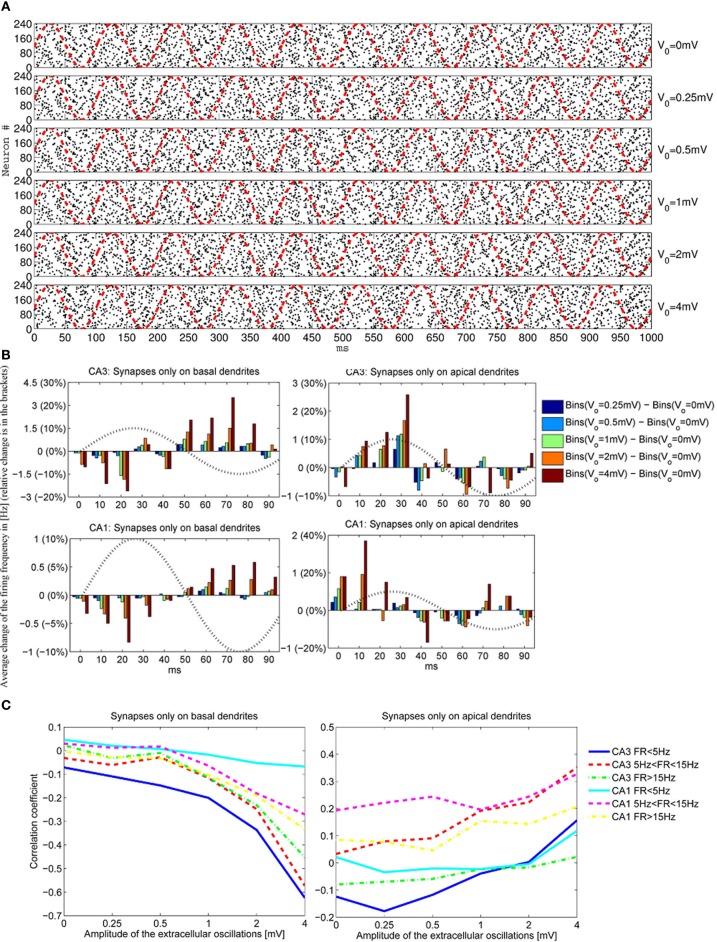
**(A)** The raster plots showing firing times of CA3 neurons for different amplitudes of the extracellular oscillations, *V*_*o*_ = 0, 0.25, 0.5, 1, 2 and 4 mV, across 48 iterations for 5 morphologies, resulting in 240 spike trains. The phase of the extracellular oscillations is given with red dashed lines. **(B)** The average (across all 240 iterations) difference between the firing frequencies with and without the extracellular oscillations. Additionally, an observation of 1 s for each iteration is cumulated and averaged in a single period of the extracellular oscillations (100 ms). All the plots are shown for the medium firing rate, i.e., 5 < *F* < 15 Hz. The sin wave, indicating the phase of the extracellular oscillations, is plotted with a dotted line and with a dimensionless amplitude. **(C)** Correlation coefficients between bins of 1 ms and the phase of the extracellular oscillations for the CA3 and the CA1 neurons for different firing rates *F*.

To have a better understanding of the correlation between the extracellular oscillations and the resulting changes in the timing and the number of action potentials, we performed an ensemble of simulations: simulations on 5 CA1 and 5 CA3 models were repeated 48 times (called *iterations*), resulting in a total number of 480 simulations. As described in section 2.3, in each of the 480 simulations the time onsets of the PSPs and the spatial locations of the synapses were randomly chosen. Figure 7A displays the raster plots obtained for the medium firing rate, 5 < *F* < 15 Hz, of the CA3 neuron models (5 cell morphologies, each used for 48 iterations, resulting in 240 simulations). Differences in the timing and the numbers of action potentials are evident, but they are difficult to quantify directly from the raster plots.

To provide a meaningful visualization of the changes caused by the extracellular oscillations, several processing steps were made. First, we created a peristimulus time histogram: the spikes were binned, that is, the time axis was divided into 10 ms bins and the spikes were gathered to obtain a spike count per bin. Then, the spike count of each bin was subtracted from the corresponding baseline spike count. The next step was to generate cumulative results, where all the bins corresponding to the same phase of the oscillations were summed. For example, when 10 Hz sinusoidal oscillations were applied during a 1 s long simulation, the spike counts at different phases of the oscillations were summed. The resulting graphs for basal and apical stimulations of the CA3 and the CA1 neural models, together with the plot of the phase of the oscillations at the soma, are shown in Figure 7B. Comprehensive figures of all the intermediate steps are provided in supplementary material.

The stimulations of the basal dendrites, on either the CA1 or the CA3 neurons, yielded a significant negative correlation for the high amplitude oscillations, *V*_*o*_ ⩾ 1 mV. On the contrary, the correlation was positive for the apical dendrites, with a smaller absolute value than the correlation observed for the basal dendrites. These trends, anti-correlation for the basal dendrites and correlation for the apical, are evident in Figure 7C where the cross-correlations between the bins and the phase of the oscillations for three different firing rates *F* are plotted.

The negative correlation during the stimulation of the basal dendrites is caused by the fact that an increase of the extracellular oscillations induces a decrease in the difference between the intracellular and the extracellular potential as written in Equation (3). Thus, the positive half-wave (with respect to the phase of the oscillations at the soma) of the extracellular oscillations drives the membrane potential away from the firing threshold, resulting in the negative correlation and vice versa.

The opposite signs of the correlation coefficients for the stimulation of the basal and the apical dendrites is a direct consequence of the asymmetrical morphology of the pyramidal neurons. As shown in Figure 3, for the spatial frequency of *f*_*s*_ = 0.8 mm^−1^, when the positive half-wave of the extracellular oscillations is around the soma, the negative half-wave will be around the apical dendrites. The negative half-wave of the oscillations facilitates the spike emission, raising the membrane potential toward the firing threshold. When the overall positive correlation is observed, it indicates that the distal oscillations have a higher influence than those around the soma. This is observed when the neuron is stimulated in the apical part of the dendritic tree, as shown on right hand side plot in Figure 7C.

To explain the difference between the absolute values of the correlation coefficients, which are higher for the stimulations of the basal dendrites, we speculate that the difference is influenced by the morphology of the pyramidal neurons: the apical dendrites are widely spread and the basal dendrites are gathered around the soma. Only a small part of the apical tree was affected by the peak of the oscillations, while most of the basal dendrites are affected by the peak. Furthermore, the soma and a nonnegligible part of the apical tree proximal to the soma were stimulated by oscillations with the phase opposite to the oscillations taking place in the distal apical dendrites.

The firing rate plays also a role in the sensitivity of the membrane potential to the influence of the extracellular oscillations. For CA3 models, it appears that when the basal dendrites were stimulated the largest change of the correlation coefficients is observed for low firing rate, then for medium and eventually for high firing rate. This result is inverted for the CA1 neurons, as illustrated in Figure 7B, suggesting that the link between the firing rate and the sensitivity to the extracellular oscillations highly depends on the properties of the ion channels. In our simulations the difference might come from the calcium and potassium conductances, since they differ in the two models.

**Figure 8:**
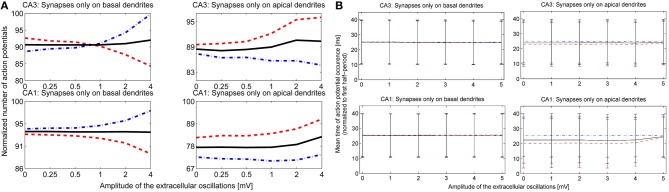
**(A) The number of action potentials as a function of the amplitude of the extracellular oscillations, with the frequency of 10 Hz and for the medium firing rate (5 < *F* < 15 Hz).** The results for the CA3 neuron models are shown in the first row and for the CA1 in the second row. The curves are obtained from results of 240 simulations (48 iterations for 5 morphologies) with 1 s duration. The results during the positive period w.r.t. the potential at the soma, are shown with red dashed lines, and during the negative period with blue dot-dashed lines and the average number of the action potentials is shown with solid black lines. **(B)** The mean timing of the action potentials with the standard deviations. The *x*-axis denotes the amplitude of the 10 Hz extracellular oscillations. To facilitate the visual comparison, we subtracted 50 ms from the negative half-wave mean timing (blue dash-dotted lines).

After observing how timing of the action potentials is influenced by the extracellular oscillations, we investigated the overall change in the number of action potentials as a function of the extracellular oscillations. This indicator is given in Figure 8A. The overall number of action potentials is computed separately for the positive and the negative period of the extracellular oscillations. The number of action potentials for the positive period is shown with red dashed lines and for the negative period with blue dot-dashed lines.

For the stimulation of the basal dendrites, both the CA3 and the CA1 neurons exhibit a decrease in the number of action potentials during the positive period and an increase during the negative period. The change is larger for the CA3 than for the CA1 neurons, suggesting that the dynamical properties of the ion channels are important for this kind of analysis. The effects are inverted for the apical dendrites and the variations are smaller than for the basal dendrites: during the positive period there is an increase in the number of action potentials, while during the negative period it decreases or does not show a significant change. This is in accordance with the previous observations where the proximal and distal contributions from the apical dendrites are balancing each other out. The average number of action potentials during both the positive and the negative period is shown with the solid black line. The average number slightly increases with the increase of the amplitude of the extracellular oscillations.

The last indicator we explored assesses the influence of the extracellular oscillations on the precise timing of action potentials. Figure 8B displays the mean timing and its standard deviation. The mean timing tends to stay near the center of the half period, which is 25 ms for the positive half-wave and 75 ms for the negative. In cases where the mean value was initially biased, i.e., significantly different from 25 or 75 ms without the extracellular oscillations, the extracellular oscillations bring the mean value closer to the middle of the half period. This is evident in the results of the stimulation of the apical dendrites in Figure 8B. This result is expected since 25 and 75 ms correspond to the peak of the amplitude of the extracellular oscillations.

#### 3.2.2. Influence of the temporal frequency of the extracellular oscillations

**Figure 9:**
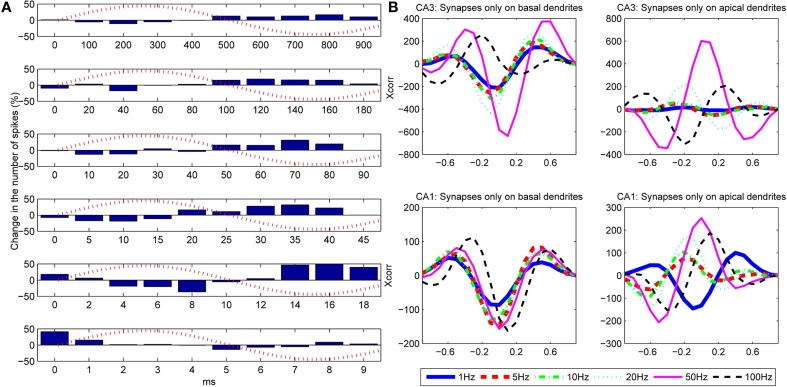
**(A)** Influence of the frequency of the extracellular oscillations on the number of action potentials for the stimulation of the basal dendrites of the CA3 neuron model. From top to bottom the plots correspond to the following frequencies: *f*_*t*_ = 1, 5, 10, 20, 50 and 100 Hz. The bins are created by cumulating the results over 1 s of simulation. **(B)** Cross-correlation between the difference of the bins (cumulated over a single period of the extracellular oscillations) and the extracellular oscillations. The cross-correlation is computed for six different frequencies of the extracellular oscillations.

To investigate the influence of the temporal frequency of the extracellular oscillations, we conducted simulations with *V*_*o*_ = 4 mV and the following set of the temporal frequencies *f*_*t*_ = 1, 5, 10, 20, 50, and 100 Hz. The results are shown in Figure 9A and displayed as histograms representing the difference in the number of action potentials caused by the oscillations. Figure 9A is given for the stimulation of the CA1 basal dendrites, and the figures that display the results of the stimulation of the CA1 apical dendrites and the CA3 basal and apical dendrites are given in the supplemental. The results are cumulated over a single period of oscillations. The phase of the oscillations at the soma is indicated with dashed red lines. The stimulation with the PSPs was set so that the firing frequency was in the medium firing range interval, i.e., 5 < *F* < 15 Hz. Spatial phase of the oscillations was $\frac{3\pi }{10}$ rad.

To evaluate the correlation between the change in the number of action potentials and the frequency of the extracellular oscillations, we computed the cross-correlation coefficient for each frequency of the oscillations. The results for the basal and the apical stimulation of the CA1 and the CA3 neurons are given in Figure 9B. The stimulation of the basal dendrites led to anti-correlation with the phase of the oscillations at the soma, which tends to be larger for higher frequencies, except for 100 Hz where it gets out of the phase. When the apical dendrites were stimulated, the relative change in the number of action potentials is still of the same order as when the basal dendrites were stimulated, but the level of correlation with the oscillations at the soma significantly varies for different frequencies. This suggests that the oscillations have important influence, not only at the soma, but at the dendrites as well.

#### 3.2.3. Influence of the spatial frequency and the spatial phase of the extracellular oscillations

**Figure 10:**
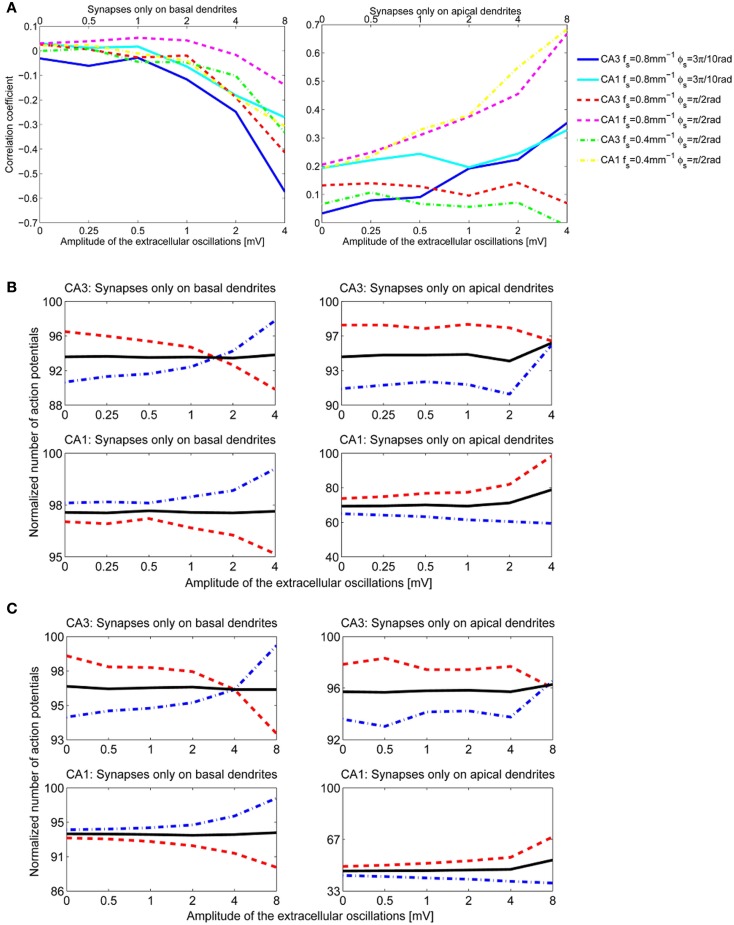
**(A)** The correlation coefficients between the bins of 1 ms width and the phase of the extracellular oscillations. Extracellular oscillations with several different spatial properties for the CA3 and the CA1 neuron models are used. **(B)** The number of action potentials as a function of the amplitude when *f*_*s*_ = 0.8 mm^−1^ and ϕ_*s*_ = $\frac{\pi }{2}$ rad. **(C)** The number of action potentials as a function of the amplitude when *f*_*s*_ = 0.4 mm^−1^ and ϕ_*s*_ = $\frac{\pi }{2}$ rad.

We performed a sensitivity analysis of the neural activity to the spatial frequency and the spatial phase of the extracellular oscillations. The methodology detailed in section 3.2.1 was applied with a different spatial frequency, *f*_*s*_ = 0.4 mm^−1^ instead of 0.8 mm^−1^ as shown in Figure 4A, and with a different spatial phase, ϕ_*s*_ = $\frac{\pi }{2}$ rad instead of $\frac{3\pi }{10}$ rad as shown in Figure 4B. The cross-correlation coefficients, computed as for Figure 7C, are shown in Figure 10A. The resulting number of action potentials is shown in Figure 10B for *f*_*s*_ = 0.8 mm^−1^ and ϕ_*s*_ = $\frac{\pi }{2}$ rad and in Figure 10C for *f*_*s*_ = 0.4 mm^−1^ and ϕ_*s*_ = $\frac{\pi }{2}$ rad. Even though the results are different for each parameter, especially for the higher amplitudes of the extracellular oscillations, the difference often appears to be rather stochastic, reflecting the highly nonlinear processing defined by the activation and inactivation functions of the active ion channels.

When ϕ_*s*_ = $\frac{\pi }{2}$ rad, the peak of the extracellular oscillations is around the soma, while for ϕ_*s*_ = $\frac{3\pi }{10}$ rad the peak occurs in the basal part of the dendritic tree (see Figure 4). The lack of significant difference between the two cases indicates that the influence of the extracellular oscillations at the soma is not more important than the influence at the dendritic tree. This suggests that for an accurate modeling of the nonsynaptic communication, the active properties of the dendritic tree should not be neglected.

The results for the apical dendrites show a great diversity, resulting mainly from the fact that the apical dendrites occupy a larger volume than the basal dendrites, thus the locations of the PSPs inputs are more spread for the apical dendrites. Also, variations across morphologies were more significant for the apical dendrites than for the basal ones, in particular for the CA1 neurons which have highly branched apical part of their dendritic tree along the entire somatodendritic axis (Spruston, 2008). This could account for the variability of the results when the stimulation is given through the apical dendrites.

## 4. Discussion

Studies that investigate how the LFPs influence timing and number of action potentials are commonly based on stimulating neurons by injection of a constant current into the soma (Parra and Bikson, 2004; Radman et al., 2007; Anastassiou et al., 2010, 2011). In this paper, we show that the neural models with active conductances and dendrites stimulated by spatially distributed alpha function-shaped excitatory PSPs (EPSPs) have highly stochastic firing dynamics, which can to some degree be influenced by the parameters of the extracellular oscillations.

The weak extracellular oscillations studied in this paper correspond to those existing spontaneously in the brain. The strength of the extracellular oscillations in the mammalian brain is commonly considered to go up to 3 mV mm^−1^. This corresponds to 1 mV oscillations in the present study. Our results suggest that these weak oscillations have a modest influence on the timing of the action potentials. Larger fields, above 6 mV mm^−1^ (which correspond to 2 and 4 mV oscillations in our study) have a significant influence on the timing. Such strong fields are seldom observed in the brain, e.g., during the hippocampal sharp waves (Buzsáki, 1986; Sullivan et al., 2011). Another source of strong oscillations could be external stimulations, for example techniques such as TES, TMS or DBS. These methods could cause significant and coherent changes in the neural activity. We speculate that inducing a synchronous neural activity in the brain using strong extracellular electric fields could help to restore the normal neural activity.

It is important to emphasize that it has been shown that small correlations, which could be induced by the extracellular oscillations, can be amplified through network interactions and lead to increase in synchronization among the neurons (Parra and Bikson, 2004; Radman et al., 2007). The neuronal synchrony has been suggested to have a computational purpose, in particular to help maintain neural rhythmic activity (Weiss and Faber, 2010).

Recent findings suggest that the extracellular oscillations of the magnitude spontaneously present in the brain usually change the membrane potential only by 1–2 mV (Radman et al., 2007; Anastassiou et al., 2010; Buzsáki et al., 2012). This is rather small with respect to the depolarization required to reach the firing threshold level from the resting potential, which is usually about 15 mV. Nevertheless, since neurons are constantly bombarded with synaptic inputs of various amplitudes, their membrane potentials are generally significantly different from the resting potential and stay in a balanced state often close to the threshold level (Wilson, 2008). When the membrane potential is close to the firing threshold, the extracellular oscillations might affect neural firing more strongly. Our results suggest that the changes in the overall firing rate are rather small, generally below 5% even for the oscillations of 4 mV amplitude. The weaker fields usually do not cause an overall change greater than 1%, see Figures 8A, 10B,C.

Changes in the firing rates show that, for a given simulation setup, the influence of the extracellular oscillations is more significant when the synaptic inputs are received through the basal than through the apical dendrites. We speculate that this is due to the spatial properties of the extracellular oscillations. The phase of the oscillations is the same along the entire basal part of the dendritic tree, as well as along the soma. On the contrary, the phase of the oscillations along the apical part of the tree is the opposite to that around the soma, thus the resulting influence of the oscillations when synaptic inputs are on the apical tree gets balanced out.

We also observed that the correlation of the firing times with the phase of the extracellular oscillations for the CA3 neuron models is stronger than for the CA1 models. This emphasizes the importance of the ion channels which have a nonlinear dependence on the voltage and alter significantly the influence of the oscillations. The presented study did not investigate all the possible scenarios through which the extracellular oscillations could have a functional role. For instance, one possible functional role could be in gating some particular (hypothetical) ion channels that could be sensitive primarily to some properties of the extracellular oscillations (e.g., voltage or slope). Such ion channels could transfer this stochastic influence of the extracellular oscillations on the membrane potential into a more structured one, which could alter the neural activity in some functional way. In our study, the CA3 model, which contained more potassium and calcium channels, was generally influenced by the extracellular oscillations in a more coherent way.

The functional role of the spontaneous LFPs is an open question, but we have shown that even if the mean firing rate is not affected, the spontaneous LFPs could influence spike timings for up to several milliseconds. This could affect the neural synchronization and thus the entire network dynamics. This is important in the context of development of biologically inspired artificial neural networks. A way to test whether an artificial neural network behaves in the same way as a biological one could be to evaluate its robustness to small changes in the spike timing.

It has been observed that the amplitude of extracellular oscillations is larger for smaller brains, i.e., it decreases from rat to cat, and from cat to primate (Buzsáki et al., 2012). One possible explanation for this is that, due to the brain size, bodies of the pyramidal neurons are vertically the best aligned in the rat brain, then in the cat brain, and in the human brain. Since studies, including this one, suggest that the amplitude of the oscillations is crucial for their coherent influence on firing properties, a decrease in the amplitude in larger brains can be an argument against the functional role of the oscillations.

### Conflict of interest statement

The authors declare that the research was conducted in the absence of any commercial or financial relationships that could be construed as a potential conflict of interest.

## Footnotes

^1^We used morphologies named: cell_a, cell_c, cell_m, cell_t, and cell_v; they are available for download on http://krasnow1.gmu.edu/cn3/L-Neuron/database/.

^2^We used morphologies named: n121CNG, n129CNG, n141CNG, n142CNG, and n145CNG; they are available for download on http://www.neuromorpho.org.

^3^Both models are available in ModelDB on the following addresses: http://senselab.med.yale.edu/modeldb/showmodel.asp?model=101629 and http://senselab.med.yale.edu/modeldb/showmodel.asp?model=55035.

^4^Within the simulator NEURON, we used mechanism extracellular to drive the voltage oscillations into each compartment.

## Supplementary material

Figure S1 **Spikes from Figure 9 in the main part of the paper binned into 10 ms time bins (only first 300 out of 1000 ms are shown).** Bins corresponding with different amplitudes of the extracellular oscillations are color coded. Phase of the extracellular oscillations at the soma is indicated with dashed black line.

Figure S2 **Difference of the time bins from Figure S1**.

Figure S3 **Influence of the frequency of the extracellular oscillations on the number of action potentials for the stimulation of the apical dendrites of CA3 neuron model**. This figure is produced in analogous way as Figure 9A in the main part of the paper. **(A)** The stimulation of the CA1 apical dendrites. **(B)** The stimulation of the CA3 basal dendrites. **(C)** The stimulation of the CA3 apical dendrites.
