# Supplementary figures and images for: Influence of extracellular oscillations on neural communication: a computational perspective
Source: Front Comput Neurosci. 2014 Feb 7;8:9. doi: 10.3389/fncom.2014.00009 (PMC3916728; doi:10.3389/fncom.2014.00009)

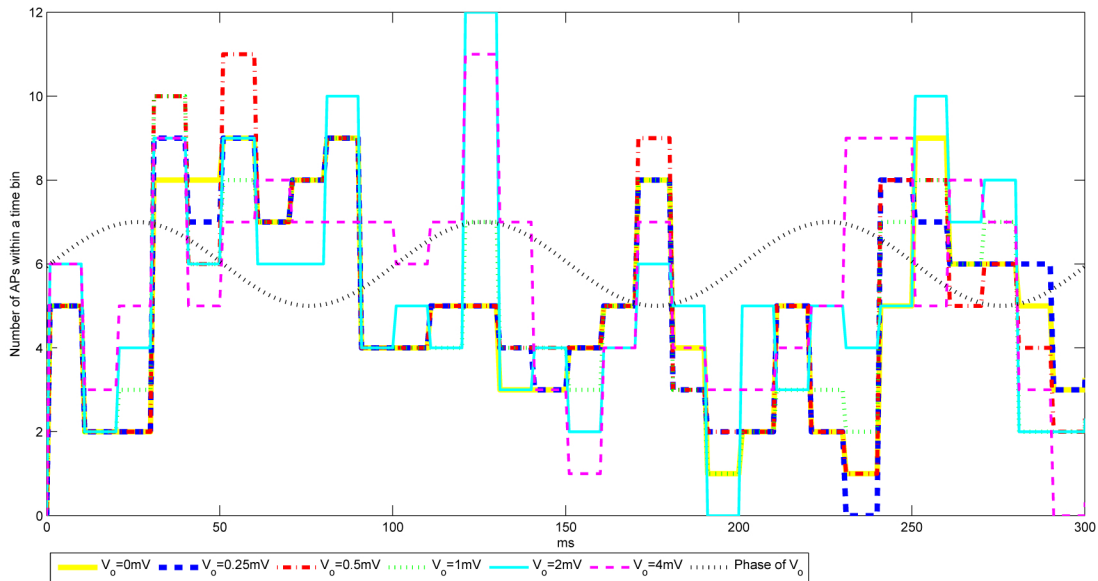

Supplement: Figure S1 — Spikes from Figure 9 in the main part of the paper binned into 10 ms time bins (only first 300 out of 1000 ms are shown). Bins corresponding with different amplitudes of the extracellular oscillations are color coded. Phase of the extracellular oscillations at the soma is indicated with dashed black line. [file Presentation1.PDF]

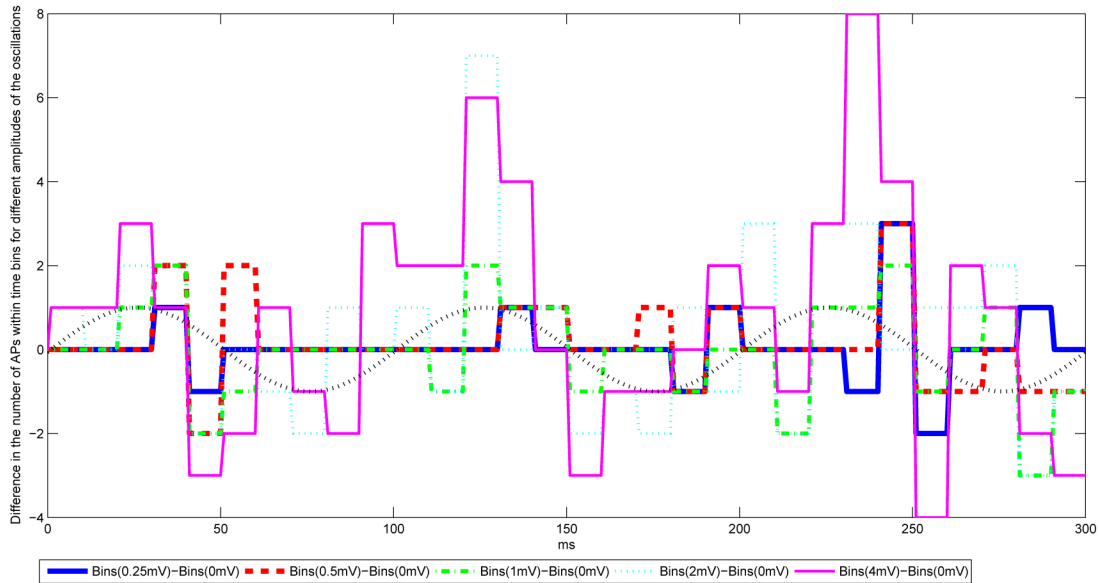

Supplement: Figure S2 — Difference of the time bins from Figure S1. [file Presentation2.PDF]

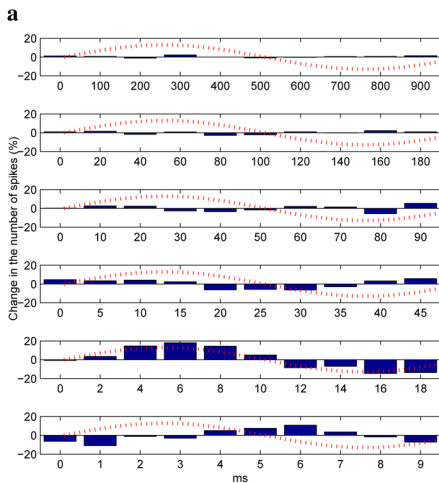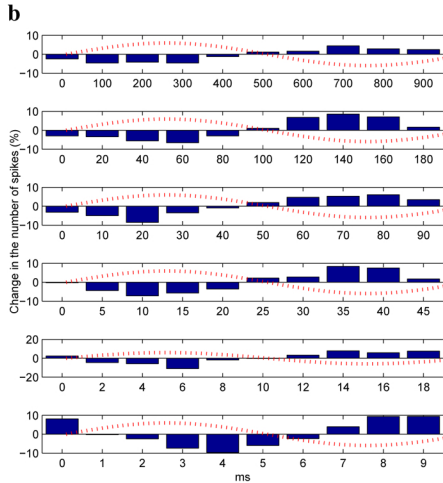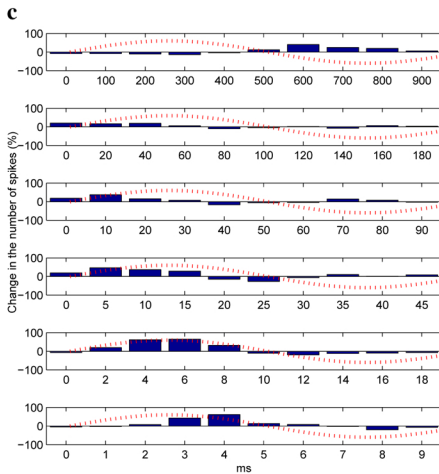

Supplement: Figure S3 — Influence of the frequency of the extracellular oscillations on the number of action potentials for the stimulation of the apical dendrites of CA3 neuron model. This figure is produced in analogous way as Figure 9A in the main part of the paper. (A) The stimulation of the CA1 apical dendrites. (B) The stimulation of the CA3 basal dendrites. (C) The stimulation of the CA3 apical dendrites. [file Presentation3.PDF]
